# Gothenburg very early supported discharge study (GOTVED) NCT01622205: a block randomized trial with superiority design of very early supported discharge for patients with stroke

**DOI:** 10.1186/1471-2377-13-66

**Published:** 2013-06-24

**Authors:** Katharina S Sunnerhagen, Anna Danielsson, Lena Rafsten, Ann Björkdahl, Åsa B Axelsson, Åsa Nordin, Cathrine A Petersson, Åsa Lundgren-Nilsson, Karin Fröjd

**Affiliations:** 1 Institute of Neuroscience and Physiology, Rehabilitation Medicine, University of Gothenburg, Gothenburg, Sweden; 2 Institute of Health and Care Sciences, University of Gothenburg, Gothenburg, Sweden

**Keywords:** Stroke, Early supported discharge, Anxiety, Recovery of function, Care giver, Longitudinal study, Participation, RCT, Qualitative interviews, Next of kin

## Abstract

**Background:**

Stroke is the disease with the highest costs for hospital care and also after discharge. Early supported discharge (ESD) has shown to be efficient and safe and the best results with well-organised discharge teams and patients with less severe strokes.

The aim is to investigate if very early supported discharge (VESD) for stroke patients in need for on-going individualised rehabilitation at home is useful for the patient and cost effective.

**Methods/design:**

A randomized controlled trial comparing VESD with ordinary discharge. Inclusion criteria: confirmed stroke, >18 years of age, living within 30 min from the stroke unit, on day 2 0–16 points on the National institute of health stroke scale (NIHSS) and 50–100 points on the Barthel Index (BI), with BI 100 then the patient can be included if the Montreal Cognitive Assessment is < 26. Exclusion criteria are: NIHSS >16, BI < 50, life expectancy < 1 year, inability to speak or to communicate in Swedish. The inclusion occurs on day 4 and in block randomization of 20 and with blinded assessor.

Primary outcome: levels of anxiety and depression. Secondary outcomes: independence, security, level of function, quality of health, needs of support in activities of daily living and caregiver burden.

Power calculation is based on the level of anxiety and with a power of 80%, p-value 0.05 (2 sided test) 44 persons per group are needed. Data is gathered on co-morbidity, re-entry to hospital, mortality and a health economic analysis. Interviews will be accomplished with a strategic sample of 15 patients in the intervention group before discharge, within two weeks after homecoming and 3 months later. Interviews are also planned with 15 relatives in the intervention group 3 months after discharge.

**Discussion:**

The ESD studies in the Cochrane review present hospital stays of a length that no longer exist in Sweden. There is not yet, to our knowledge, any study of early supported discharge with present length of hospital stay. Thus it is not clear if home rehabilitation nowadays without risks, is cost effective, or with the same patient usefulness as earlier studies.

**Trial registration:**

ClinicalTrials.gov NCT01622205

## Background

Stroke is a life-altering event that affects a large number of individuals throughout the world; one in six people worldwide will have a stroke in their lifetime. Based on data from year 2005, global estimates of stroke incidence indicate that 16 million people worldwide experience first-time strokes each year, resulting in 5.7 million deaths ( [1]). The consequences of stroke are significant and severe: the disorder is a leading cause of physical disability among adults globally ( [1,2]). Stroke is the disease with the highest costs for hospital care and also for care and rehabilitation after discharge, which both affects the person’s and caregiver’s quality of life and also the expenses for the society. Initiatives that could optimize rehabilitation for stroke patients are important. Multi professional team-based person-centred care is the most important factor for success in stroke rehabilitation ( [3,4]). However, the timing and content of the rehabilitation needs to be further analysed.

Early rehabilitation ideally begins in the inpatient settings, immediately after a diagnosis of stroke has been confirmed and the patient is stabilized. Here, the priority goals are patient mobilization, prevention of secondary stroke and stroke-related sequel, encouragement of self-care, provision of emotional support and education, and assessment of general health. With their resources focused on the acute phase of stroke, stroke units may discharge patients with inadequate early rehabilitation services ( [5]). All too often this practice of “pushing” patients from one care environment to the next, particularly from the acute-care phase to the early rehabilitation phase, leaves patients and caregivers responsible for navigating an unfamiliar health system to effectively arrive at the next appropriate phase of care. Such philosophies of patient management are often dictated by third-party payers and allocation of health resources. Patients and caregivers have identified a significant lack of a seamless, integrated continuum from acute care onward as a major obstacle to navigating the rehabilitation process ( [5]).

Rehabilitation concerns optimizing the levels of function, activity and the persons own participation in hers/his context. A multidisciplinary approach is needed in complex situations, as after a stroke, and the rehabilitation must be individualised according to personal goals. The rehabilitation begins in the stroke care unit, but needs to go on for months and sometimes years if the person will regain optimal levels of function and activity. In Sweden the primary health care and the local government are responsible for the basic rehabilitation after a hospital stay. However, there are indications of lack of competence for stroke rehabilitation and the rehabilitation is rarely based on multidisciplinary team that work integrated. Therefore, many stroke patients don’t get optimal rehabilitation which can be seen in the report from the Swedish stroke quality register, where only about 60% of the patients in Gothenburg are satisfied with the support of their needs after discharge ( [6]). This indicates that there might be a need for some sort of case manager for the person in the rehabilitation process after stroke, which supports the person in contacts with primary care, rehabilitation units, home help service etc.

Early supported discharge (ESD) has shown to be efficient and safe in different setting ( [7,8]). The persons who were sent home earlier with support were more likely to remain at home in the long term and to regain independence in their daily activities. They also reported higher degree of satisfaction with the rehabilitation ( [7]). The best results were seen with well-organised discharge teams and patients with less severe strokes. From Stockholm, it has been shown that till 5 years after stroke, the ESD service was favourable with regard to resource use ( [9]).

In Sweden, supported training in the home setting has been suggested to be a bridge between stroke unit care and the health care of the primary care organisation and the local government. Home rehabilitation as early supported discharge of stroke patients is recommended (priority 3) in the Swedish National Guidelines of stroke 2009: ”Stroke patients with mild to moderate remaining symptoms who gets early supported discharge from the stroke care unit of a multidisciplinary team for home rehabilitation, have shorter hospital visit, less severe dependence of others for activities of daily living (ADL) and are more satisfied with the care” ( [10]). The home rehabilitation intervention in the referred studies resulted in shorter hospital stay (median 8 days). However, the control group had a hospital stay of 33 days and the intervention (early supported discharge) group 25 days. A Cochrane review showed that the cost for the care was 9 to 20% lower in the intervention group ( [7]). A consensus on the content in early supported discharge from the trialists came up with the recommendation that multidisciplinary, specialist stroke ESD teams should plan and co-ordinate both discharge from hospital and provide rehabilitation in the community ( [11])

The studies in the Cochrane review ( [7]) present hospital stays of a length that no longer exist in Sweden. The average hospital stay after acute stroke, is 15 days (with a median of 8) according to the Swedish quality register, thus very much shorter than in the referred studies ( [6]). There is not yet, to our knowledge, any study of early supported discharge with present length of hospital stay. Thus it is not clear if home rehabilitation nowadays without risks, is cost effective, or with the same patient usefulness as earlier studies. A randomised study of early supported discharge for stroke patients in need for on-going individualised rehabilitation at home can reveal if the intervention is useful for the patient and cost effective.

The aim is to investigate if very early supported discharge for stroke patients in need for on-going individualised rehabilitation at home due to motor and/or cognitive dysfunction is useful for the patient and cost effective. The intervention should be person-centered ( [12]); an approach based on who the person is: their context, their history, their family and loved ones, their individual strengths and weaknesses.

### Hypothesis and expected results

The main hypothesis is that the intervention will lead to less distress for the person with stroke. As secondary hypothesis, the assumption is that the distress will be less for the next of kin as well as result in better or likewise motor and/or cognitive function, at one, three and twelve months after the stroke.

The expectation is that the results will show that early supported discharge is medically safe for the patient. The patients and the relatives will feel more secure after discharge from the stroke care unit and thereby the patients perceive less depression, higher levels of function and activity and better health quality. The next of kin might experience less caregiver burden. Another aim is to analyse the participation of the patient in the rehabilitation process.

Further, the expectation of the qualitative interviews is that they will reveal how the patient and the next of kin experience the home rehabilitation and possibilities and obstacles to patient and care giver’s participation in the rehabilitation.

## Methods/design

### PICO

Patient/PopulationPersons with stroke at a stroke unit

InterventionVery early supported discharge to the home

ComparisonOrdinary discharge to the home

OutcomeReduction in anxiety, same amount of re-admissions and the same level of improvement in motor skills, ADL function and cognition as for the control group

### Design and population

The design is a randomized controlled trial with blinded assessors. The study population is the stroke patients at the Sahlgrenska stroke care unit. The Stroke unit at Sahlgrenska University Hospital serves the larger Gothenburg urban area, thus all persons from this catchment area are randomly referred to the Sahlgrenska University Hospital. The project is approved by the Regional Ethical Review Board in Gothenburg http://www.epn.se/sv/goeteborg/om-naemnden/ and the Helsinki declaration is followed. Written informed consent will be obtained from the participants or from their closest relative.

Primary outcome levels of anxiety and depression. Secondary outcomes are independence, security, level of function, quality of health, needs of support in ADL and caregiver burden.

Data entered the Riks-stroke database is used for back-ground information of each patient as well as stroke type, location, length of stay etc. Data on support and training given after discharge as well as home modifications during the first year will be gathered.

#### Assessments (all translated into Swedish)

Body function:

•Montreal Cognitive Assessment (MoCA) ( [13]),

•The MoCA has been proposed as a screening tool that promises good sensitivity to deficits arising from stroke and vascular cognitive impairment The MoCA includes sections on visuospatial/executive, naming, attention, language, abstraction, delayed recall, and orientation. It is scored out of 30 (extra point for <13 years' education) and the recommended “normal” cut-off is ≥26.

•modified Rankin Scale (mRS) ( [14]),

•The mRS is used to describe overall disability. The scale runs from 0–6, running from perfect health without symptoms to death.

•Fugl-Meyer sensory motor assessment (FMA) ( [15])

•Fugl-Meyer Assessment, Upper (maximum score of 66 corresponds to normal motor function) and Lower extremity (maximum score of 34). The non-motor domains of FMA sensation, passive range of motion and pain during passive joint motions will be completed as well.

•Figure of 8 for the hand

•To assess hand volume on both hands using an ordinary tape measure (cm).

Activity level

•Time up and go (TUG) ( [16]),

•The TUG is testing basic mobility. The time (s) required for an individual to stand up from a standardized chair, walk a distance of three meters, turn, walk back to the chair and sit down again. A shorter time indicates better performance. The has been shown to be reliable and valid in this group of patients

•Berg Balance Scale (BBS) ( [17]),

•The BBS assesses functional balance. Performance in this test is scored from 0 (cannot perform) to 4 (normal performance) on 14 different tasks, including ability to sit, stand, reach, lean over, turn around and step. The maximum score on the BBS is 56. A higher score indicates better balance skills

•ADL taxonomy ( [18]),

•The ADL taxonomy is used for the investigation of ADL task performance and includes 12 activities. The first seven activities deal with the Personal ADL and the other five, with Instrumental ADL. During each activity are also a number of subtasks that are ranked by difficulty and can be judged on various aspects such as independence, dependent or not relevant.

•Barthel index (BI) ( [19])

•The BI measures the extent to which somebody can function independently and has mobility in their ADL i.e. feeding, bathing, grooming, dressing, bowel control, bladder control, toileting, chair transfer, ambulation and stair climbing. Each performance item is rated on this scale with a given number of points assigned to each level. In this study the modified version with 0–100 is used, where a lower score indicates dependency.

Participation level:

•Stroke Impact Scale (SIS) 3.0, ( [20]),

•Stroke Impact Scale is a questionnaire on different aspects of the stroke recovery where the person replies on their perception regarding their life after the stroke. The 59 questions are divided into 8 domains; strength, memory, emotion, communication, activities of daily living, mobility, hand function and social participation.

•Impact on Autonomy and Participation, English version (IPA-E) ( [21]),

•IPA-E is a generic outcome measure for adults with chronic conditions where the person estimates perceived limitations in participation and autonomy related to dependency in the current living surrounding. The subscales include autonomy indoors, family role, autonomy outdoors, social life and relationships, work and education. Additionally, IPA-E identifies the extent to which limitations in life are experienced as problematic in areas of mobility, self-care, activities, economy issues, social life, work and education. IPA-E is valid, reliable and sensitive to change after stroke.

Environmental level:

Caregiver Burden Scale (CBS) ( [22]),

The CBS is a questionnaire with 22 questions (answered in written by the carer) concerning burden from the aspects of the caregiver’s health, feeling of psychological well-being, relations, social network, physical workload and environmental aspects that might be important. When the scale was developed, factor analysis was used to yield five indices – general strain (8 questions), disappointment (5 questions), isolation (3 questions), emotional involvement (3 questions) and environment (3 questions). The items are scored from 0 to 3 (Not at all, Hardly, Somewhat and Definitely), maximum score 66. (Caregiver burden could be viewed as an environmental factor)

*Patient reported outcomes (not classified according to the ICF)*:

•Hospital Anxiety and Depression Scale (HADS) ( [23]),

•The HADS is a 14-item questionnaire that consists of anxiety (HADS-A) and depression (HADS-D) subscales. It has good psychometric properties and is well suited for assessing symptom severity in stroke patients. A HADS-Total score of greater than 10 indicates clinically significant emotional distress.

•Falls efficacy Scale (FES) ( [24]),

•The FES assesses perceived self-confidence in ADL-task performance without falling. It consists of 13 items including personal activities of daily living, items 1–6 and instrumental activities of daily living, items 8–13 and item 7, assessing the ability to walk up and down stairs. Confidence in performing each activity is rated on a visual numeric scale ranging from 0, not at all confident, to 10, completely confident, giving a possible maximal score of 130, and a total score of 60 for each of the two subscales.

•EuroQol Quality of Life Scale (EQ-5D) ( [25]),

•EuroQol Quality of Life Scale is a widely used generic measure and includes five dimensions: mobility, self-care, usual activities, pain/discomfort and anxiety/depression. The EQ-5D includes a visual analogue scale on which the patients rate their own health between 0 and 100.

Inclusion criteria

Confirmed stroke according to WHO´s criteria

>18 years of age

Living within 30 min from the stroke unit

On day 2 National institute of health stroke scale (NIHSS) ( [26]) of 0–16 points and BI 50–100 points.

MoCA index < 26 if BI = 100.

Inclusion criteria

NIHSS >16

BI < 50

Life expectancy < 1 year (as with severe malignancy)

Does not speak or communicate in Swedish prior to the incidence

A power calculation has been performed based on the level of anxiety (assessed with the HADS) in patients at stroke units ( [27,28]) and also what has been judged as problematic level of anxiety and long term outcome after stroke ( [29]). The assumption is that some anxiety is normal but the aim is to reduce the risk of problematic anxiety with the intervention. With a power of 80%, p-value 0.05 (2 sided test) 44 persons per group are needed. The aim is therefore 55 persons per group, since deaths may occur as well as withdrawn consent; a total of 110. The investigation also includes data of co-morbidity, re-entry to hospital and mortality. Furthermore, data from the Swedish quality register of stroke including information of ADL function before stroke, presence of follow-up visits and questions regarding the subjective experience from the rehabilitation. Data also include information about how satisfactory the information about stroke and where to get support has been as well as the patients view concerning the sufficiency of assistance and support from home care or next of kin. Data of the amount of granted home help service from the local authority will also be collected for health economic analysis as performed by Lundström et al. ( [30]).

Interviews will be accomplished with a strategic sample of 15 patients in the intervention group before discharge, within two weeks after homecoming and 3 months later to describe the patient’s subjective expectations and experiences of early discharge and rehabilitation. Interviews are also planned with a sample of about 20 relatives (caregivers) in the intervention group 3 months after discharge to describe their subjective experiences of early discharge and rehabilitation from the perspective of caregivers. In both cases, interview guides with question areas will be used. The interviews will be recorded and transcribed verbatim and further analysed with qualitative content analyses according to Krippendorff ( [31]).

The planning of the home rehabilitation with the patient together with the team will be studied to analyse possibilities and/or obstacles for patient participation and activity in this process. Data will be gathered by digital audio recording of 10 planning meetings and note-taking and analysed with qualitative content analyses.

Further, observation through participation will be used to study the interaction between the patient and the team when the person is at home and how the patient participates in the rehabilitation. Data will be gathered by video recording and note-taking and analysed with both a quantitative and a qualitative approach.

Time schedule of data gathering

Day 2: (36–60 hours)

BI and MoCA performed by the occupational therapist

Day 4: (+/− 1 day)

Information about the study and randomization after informed consent

Demographic data will be collected during the first assessment. Stroke subtype will be confirmed by imaging. Ischemic strokes will be classified for subtype and site for lesion by using TOAST and Bamford classifications( [32]). Treatments of thrombolysis or thrombectomy will be registered. Additional data will be extracted from the national quality register for stroke - Swedish Stroke Register.

Day 5: (+/− 1 day)

•Assessment 1

Assessment 1 (HADS, SIS, MoCA FMA, mRS)

Day 5–10 Discharge planning

Day 5–10 Discharge (First interview)

Within 24 hours after discharge: TUG, BB, ADL taxonomy, BI, HADS, EQ5D and FES.

Within two weeks after discharge: Second interview

Assessment 2 (1 month) ADL taxonomy, BI, BT, TUG, HADS, EQ5D, FES and mRS.

Next of kin: HADS

Assessment 3 (3 months) ADL taxonomy, BI, BB, TUG, HAD, EQ5D and FES, SIS, mRS and IPA-E.

Third interview with patients and interview with relatives.

Next of kin: HADS and CBS

Assessment 4 (12 months) BI, BB, TUG, HAD, EQ5D, FES, SIS, mRS and IPA-E. FMA and MoCA.

Next of kin: HADS and CBS

#### Discharge planning at the stroke unit

The stroke nurse has the responsibility during the in-patient time to communicate with the patient in order to understand the experience of the stroke, which, together with the objective signs of the stroke, is the base for discussions and the planning of the care with the patient together with the team. The nurse is also responsible for the discharge planning, the co-ordination of the rehabilitation and the contacts with primary health care. The nurse also co-ordinates the contacts with the next of kin and involves them in the planning. If the patient and nurse have identified the need for home help service, the nurse also makes these contacts. Patients with need for speech therapy after discharge will receive this at a speech therapy out-patient clinic. Before discharge the patient, the VED team and next of kin explore the patient’s needs and wishes and decide on individual goals for the intervention period.

#### Intervention in the very early supported group

A rehabilitation team made up of physiotherapists, occupational therapists and a stroke nurse from the stroke care unit continues the rehabilitation in the patient’s home. The intervention has a person-centered approach which is based on who the person is: their context, their history, their next of kin, their individual strengths and weaknesses ( [12]). Goal setting using questions as in the Canadian Occupational Performance Measure ( [33]) takes part before the discharge. Examples of goals can be: to be able to go to the local store to buy milk, to be able to hang the laundry or to be able to travel on the tram to the daughter or how to manage the bills.

At discharge, the intervention group will get an individual schedule for the first week of home rehabilitation. The intervention comprises 2–4 visits per week of the home rehabilitation team members and one to two stroke nurse’s visits. The intervention may imply training in different activities or to think through different ways to adapt in difficult situations in order to achieve a goal. For some patients the intervention may be to try the pursued activities with safe support so that they may feel secure in their performance.

The patient and the home rehabilitation team decide when the supported discharge should end, with a maximum of four weeks after discharge. During this period contacts will be made with those who will carry on the rehabilitation afterwards. Important for the period is also information and support to next of kin and home care service on how to best support the patient. Speech and language therapy will be delivered if needed.

#### Control setting after discharge

The control group will have support as usual after discharge e.g. if needed home help service, outpatient rehabilitation of a physiotherapist and or an occupational therapist or speech and language therapy.

### Time plan

The project started in May 2011 and recruitment is estimated to take 3 years.

### Significance

There are at least three different perspectives of significance in the project; the personal, the societal and the public health.

From the personal perspective, to get back home as soon as possible and to be able to participate in the planning of the rehabilitation and decide the goals should results in empowerment and hopefully better satisfaction with the stroke care. Less anxiety increases the chance of well-being.

From the societal perspective, the possibility that it is safe to discharge patients early with support from the stroke unit means that money will be saved. If the family feels less distressed when support is given in the home, the risk for re-admittance as well as stress induced symptoms in the next of kin should be reduced. This is also a chance to enhance quality for the family of a stroke survivor and perhaps reduce the risk of ill health for the care giver.

From the public health perspective, the chance to reduce anxiety and support the next of kin in the large population of stroke survivors should have a high priority. The relatives often today report a high caregiver burden.

The expectation is to show that early supported discharge is safe. The patients and the relatives will feel more secure after discharge from the stroke care unit and thereby the patients perceive less anxiety and depression, higher levels of function and activity and better health quality.

### Preliminary results

A pilot study at started in April 2010 in order to evaluate a model of early supported discharge. The overall goal for the project was to improve the quality of the care at home after discharge from the stroke care unit. Stroke patients in need for on-going training at home of motor and/or cognitive dysfunction have been included in the study. The patients, the relatives and the involved rehabilitation staff in the pilot study have been mostly overwhelmingly positive to the supported discharge. The patients in the pilot project received 3–19 visits at home (on average 9 occasions). The division of labour between the professions depending on needs and goals resulted in1.6 visits by the nurses, 3.4 visits by the occupational therapist and 4.3 by the physical therapist. The support at home seems to make the patients and the relatives more secure and the patients seems to have a better chance to regain activities and functions with home rehabilitation.

The current project started in May 2011 with inclusion for 3 weeks prior to the vacation period and continued in September. So far, 40 patients have been included.

### Ethical considerations

The study has ethical approval (110221, 042–11).

There is an ethical problem that a practice of early discharge is developing in Sweden that is not evidence based and not always quality controlled. Support in the home is sometimes given from a team from a stroke unit, sometimes from primary care and often from home care personnel. Therefore, the ethical problem here deals mainly with assessing patients and caregivers many times, where the results of the assessment not necessarily will be of benefit for the assessed person. Qualitative research with interviews of the patient and the next of kin where areas such as having influence of the care/rehabilitation will be discussed may yield discomfort. In order to minimize this, the interviewer will be from outside ordinary care at the hospital and well trained in qualitative research.

## Discussion

The ESD studies in the Cochrane review present hospital stays of a length (33 days) in the control group that no longer exist in Sweden. The average hospital stay after is 15 days. There is not yet, to our knowledge, any study of early supported discharge with present length of hospital stay. Thus it is not clear if home rehabilitation nowadays is without risks, is cost effective, or with the same patient usefulness as earlier studies. The research on emotional and practical support in the home after discharge gives conflicting results and the scientific evidence is insufficient. The reason for choosing level of anxiety as primary outcome is that this is a common problem after stroke and probably can be affected by having support in the home setting. The length of stay will probably vary very little between the groups and the training efforts in the intervention group might not necessarily show in the areas of body function or ADL.

## Abbreviations

(ADL): Activities of daily living; (BI): Barthel index; (BBS): Berg Balance Scale; (CBS): Caregiver Burden Scale; (ESD): Early supported discharge; (EQ-5D): EuroQol Quality of Life Scale; (FES): Falls efficacy Scale; (FMA): Fugl-Meyer sensory motor assessment; (HADS): Hospital Anxiety and Depression Scale; (IPA-E): Impact on Autonomy and Participation; (MoCA): Montreal Cognitive Assessment; (mRS): modified Rankin Scale; (NIHSS): National institute of health stroke scale; (SIS): Stroke Impact Scale; TOAST: Trail of Org 10172 in Acute Treatment; (TUG): Time up and go.

## Competing interests

The authors declare that they have no competing interests.

## Authors’ information

All authors affiliated with the Gothenburg University Center for Person-centered Care http://www.gpcc.gu.se/english/

## Authors’ contribution

KF, LR and AB were involved in the pilot study. KSS designed the large trial together with KF, LR and AB with input from ÅLN and AD. CP and AB specifically looked into the outcome measures for occupational therapy, AD and LR at the outcome used for balance. KSS and ÅA were responsible for the qualitative part of the study with input from ÅN. KSS were financially responsible. All authors read and approved the final manuscript.

## Pre-publication history

The pre-publication history for this paper can be accessed here:

http://www.biomedcentral.com/1471-2377/13/66/prepub
